# Evaluating complete remission with partial hematologic recovery (CRh) as a response criterion in myelodysplastic syndromes (MDS)

**DOI:** 10.1038/s41408-022-00748-9

**Published:** 2022-11-15

**Authors:** Andrew M. Brunner, Alexander Gavralidis, Najla Al Ali, Anthony Hunter, Rami Komrokji, Amer Zeidan, David A. Sallman

**Affiliations:** 1 Massachusetts General Hospital Cancer Center, Boston, MA USA; 2 North Shore Medical Center, Salem, MA USA; 3 Moffitt Cancer Center, Tampa, FL USA; 4 Emory University, Atlanta, GA USA; 5 Yale University School of Medicine, New Haven, CT USA

**Keywords:** Myelodysplastic syndrome, Phase I trials

## Abstract

Myelodysplastic syndromes (MDS) treated with DNMTI therapy have responses according to the 2006 IWG response criteria. CR responses have had the strongest association with OS. Recently, CR with partial hematologic recovery (CRh; i.e. blasts <5%, ANC > 500, platelets > 50) has been evaluated in AML, but its relevance is unknown in MDS. We identified adult patients with MDS treated with DNMTIs. We assessed best overall response to therapy according to IWG 2006 criteria, and subsequently identified patients meeting CRh criteria from the subgroup with SD or mCR. We evaluated duration of therapy and overall survival according to response. We identified 311 patients with MDS who received treatment between 2007 and 2018. The median age at the time of therapy was 69 years (range 23–91). Median follow up was 60 months. According to IWG 2006, responses included CR (*n* = 43, 14%), PR (*n* = 2, 1%), mCR (*n* = 57, 18%), SD (*n* = 149, 48%) and PD (*n* = 60, 19%). 79 patients (25%) achieved HI. A total of 62 patients (20%) met CRh criteria leading to reclassification of mCR (now *n* = 26, 8%) or SD (now *n* = 118, 38%). Patients achieving CR had similar time on therapy (median 8.1mo) compared to CRh (median 6mo, HR 1.4, 95% CI 0.9–2.0), and longer than other responses (*p* < 0.001). OS varied according to response; median OS was similar between CR (23.3mo) and CRh (25mo, HR 1.28 [0.79–2.08]), which was longer than those with mCR (17.2mo, HR 1.71 [0.96–3.05]), SD (16.3mo, HR 1.61 [1.04–2.48]), and PD (8.7mo, HR 3.04 [1.91–4.83]) (*p* < 0.001). OS associations with CR/CRh were confirmed in multivariable analysis accounting for allogeneic transplant. MDS patients who achieve a CRh response had similar survival and duration on therapy as patients who achieve CR response and superior to other IWG responses. These data support further evaluation of CRh into future response criteria and clinical trials.

## Abstract (web summary)

Following treatment with DNMTI therapy, patients with MDS have differences in survival according to the quality of their treatment response. In this analysis, patients who achieve CRh had similar OS compared to those meeting standard “CR” criteria, which was better than the survival of those with other treatment responses (*p* < 0.0001).

## Graphical abstract

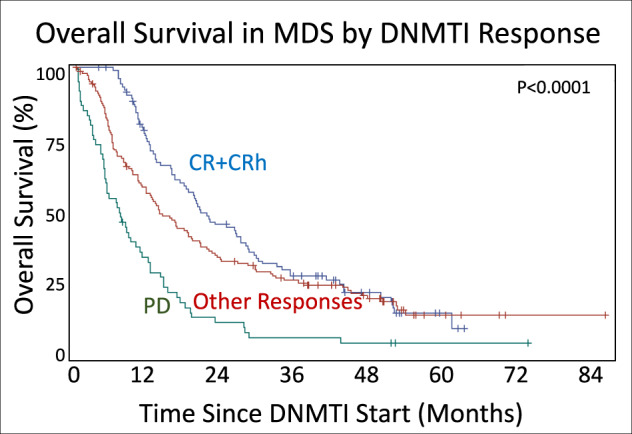

## Introduction

Myelodysplastic syndromes (MDS) are characterized by ineffective malignant hematopoiesis and an increased risk of progression toward more advanced myeloid neoplasia, acute myeloid leukemia (AML) [1, 2]. Complications in MDS are typically related to the sequelae of this ineffective hematopoiesis – a risk of infection, bleeding, and complications of anemia [3]. Therefore, a common underlying goal of chemotherapeutics utilized in MDS is not only to prolong survival, but also to improve blood counts and limit the complications associated with cytopenias.

Unfortunately, while currently available treatments may provide symptomatic improvement, and some are associated with improvements in survival, none are curative without allogeneic transplant [4–6]. DNA methyltransferase inhibitors (DNMTIs, also termed hypomethylating agents or HMAs) have been shown to prolong survival in MDS compared to other therapeutics including chemotherapy and best supportive care [7–10]. Assessing the response to DNMTI therapy can be challenging not only because of the significant variation of baseline blast and blood counts among patients with MDS, but also due to significant heterogeneity in bone marrow blast response and hematologic responses during treatment [11].

Trials evaluating the activity of DNMTI therapy in MDS have typically utilized a set of standardized response criteria proposed by an international working group (IWG) in 2000, and subsequently revised in 2006 and 2018 with the latest iteration most relevant to lower-risk MDS patients and blood cell transfusion dependence [12–14]. These response criteria comprise bone marrow blast responses and whether or not complete blood count recovery is present—and separately describe hematologic lineage specific responses. The IWG response criteria have been validated in the setting of DNMTI therapy in MDS, in that achieving specific responses with DNMTI therapy have been associated with improvement in overall survival (OS) [15, 16]. Notably, the achievement of complete remission (CR) has the strongest correlation with improved overall survival while hematologic improvement (HI) has modest correlation with improved OS, and patients with marrow CR (mCR) alone have no improvement [15, 17].

It is not clear to what degree other responses may also be associated with survival and therefore may be meaningful outcomes with prognostic significance [11]. The 2006 IWG response criteria include CR, partial remission (PR), marrow complete remission (mCR), stable disease (SD), and progressive disease (PD), as well as separate assessment of hematologic improvement (HI) [13]. The distinctions between these response criteria can be somewhat arbitrary – for instance, CR requires a hemoglobin of 11 g/dL, although it is not clear whether such a response is truly better than a patient who otherwise meets CR criteria and is transfusion independent but has a hemoglobin of 10 g/dL. Additionally, the standard of care for higher risk MDS patients is indefinite DNMTI therapy and thus vacillation of counts, particularly around time of disease assessments, is significant where ANC, platelets and hemoglobin may at times be consistent with CR although may intermittently drop below the required thresholds.

Recently, CR with partial hematologic recovery (CRh, blasts <5%, ANC > 500, Plt >50) has been proposed as a response criteria for patients with acute leukemias, including acute lymphoblastic leukemia and acute myeloid leukemia, and when paired with measurable residual disease testing it represents both disease control (blast reduction <5%) and clinically relevant count recovery (ANC > 500/μL, platelets >50 k/μL) [18]. Importantly, many prospective clinical trials in AML have primary endpoints using a composite CR/CRh metric given the clinical benefit to patients, and several therapies have been approved based on these responses. Although measurable residual disease is less defined in MDS [19], CRh describes a combined marrow/blood metric or “goal” with relevance to MDS patients and their comorbid cytopenias. The relevance of CRh as an outcome metric is unknown in MDS, and exploring CRh may serve to also explore other novel response criteria in MDS clinical trials, ideally leading to future approved therapies for patients. We therefore sought to identify patients with MDS treated on DNMTI therapy whose response could be reclassified as CRh, and to understand the clinical characteristics and patient outcomes of this cohort.

## Methods

We retrospectively identified a cohort of adult patients age 18 and older, who were diagnosed with MDS with fewer than 20% blasts, and who were treated with either azacitidine or decitabine at Massachusetts General Hospital and Moffitt Cancer Center. Patients were followed from the time of treatment initiation until death or last known alive (LKA). We retrospectively assessed treatment responses during the period of treatment with azacitidine or decitabine. When clinical assessments were available in chart review, these were confirmed and utilized if they met IWG criteria.

We initially assessed the best overall response during the period of DNMTI therapy according to IWG 2006 criteria, assigning the best overall marrow response as CR, partial remission (PR), mCR, stable disease (SD), or progressive disease (PD). We also assessed hematologic improvement by lineage (erythroid, myeloid, neutrophil). Subsequently patients were reviewed to determine whether they met CRh criteria (bone marrow blasts <5%, ANC > 500 cells/μL, Plt >50 k/μL), and recoded marrow responses according to these modified criteria (CR, PR, CRh, mCR, SD, PD). This resulted in the reclassification of a portion of patients who previously met the criteria for SD or mCR into the group meeting CRh. We then compared outcomes according to each of these defined cohorts.

We assessed patient and disease characteristics that were associated with those patients achieving CRh compared to other responses using Chi-square/Fisher exact testing and Wilcoxon testing, as appropriate. We evaluated the duration of HMA therapy according to these modified response criteria, defined as the time of HMA start until treatment cessation. We also assessed overall survival according to response, starting at the time of treatment start, and until death or censored at last known alive using the method of Kaplan and Meier. Multivariate analysis of survival estimate was performed using Cox regression, with allogeneic transplant included as a time-varying covariate by the method of Fine and Gray.

## Results

We identified a total of 311 patients with MDS who received treatment between 2007 and 2018 (Table 1). The median age at the time of HMA therapy was 69 years (range 23–91). The median ANC was 1.3 (range 0–22.3), hemoglobin was 9.5 (4.0–14.0), and platelets were 76,000 (7–868 k). The median percentage of bone marrow blasts were 5% (0–19%). When evaluating patients according to disease risk as assessed by WHO and IPSS-R scores, the majority of patients had higher risk disease features, with most harboring excess blasts (*n* = 181; 58%) and 71% with intermediate or higher risk MDS by IPSS-R (Table 1). The majority of patients identified were treated with azacitidine chemotherapy (*n* = 238, 77%). Median follow-up was 60 months.

**Table 1:** Patient characteristics.

	All patients (*n* = 311)	Patients with CR (*n* = 43)	Patients with CRh (*n* = 62)	Patients with SD/mCR (*n* = 144)	*p* value
Age	69 (23–91)	70 (58–86)	69 (45–86)	69 (23–91)	0.20
Sex (male)	203 (65%)	30 (70%)	39 (63%)	92 (64%)	0.77
Blood counts					
ANC	1.24 (0–22.3)	0.8 (0.1–4.1)	1.8 (0–5)	1.2 (0.1–10.8)	0.003
Hgb	9.5 (4–14)	10.2 (8.4–13.8)	9.6 (4–12.8)	9.2 (5.3–13.8)	0.04
Platelets	76 (7–868)	81 (14–271)	104 (7–415)	71 (8–558)	0.03
BM blasts	5% (0–19%)	7 (0–19)	3.5 (0–16)	5.5 (0–17)	0.01
WHO category					0.16
MDS del(5q)	3 (1%)	0	0	3 (2%)	
MDS SLD	19 (6%)	3 (7%)	4 (6%)	10 (7%)	
MDS MLD	95 (31%)	9 (21%)	28 (45%)	44 (31%)	
MDS EB1	100 (32%)	19 (44%)	18 (29%)	43 (30%)	
MDS EB2	81 (26%)	11 (26%)	9 (15%)	41 (28%)	
MDS NOS	13 (4%)	1 (2%)	3 (5%)	3 (2%)	
IPSS-R risk					0.22
Very low	21 (7%)	4 (9%)	4 (7%)	8 (6%)	
Low	65 (22%)	10 (23%)	19 (34%)	26 (18%)	
Intermediate	67 (22%)	8 (19%)	10 (18%)	41 (29%)	
High	59 (20%)	7 (16%)	13 (23%)	27 (19%)	
Very high	87 (29%)	14 (33%)	10 (18%)	40 (28%)	
Treatment					0.66
Azacitidine	238 (77%)	32 (74%)	49 (79%)	116 (81%)	
Decitabine	73 (23%)	11 (26%)	13 (21%)	28 (19%)	
Allogeneic transplant					
Yes	106 (34%)	16 (37%)	29 (47%)	48 (33%)	0.19

**Fig. 1:**
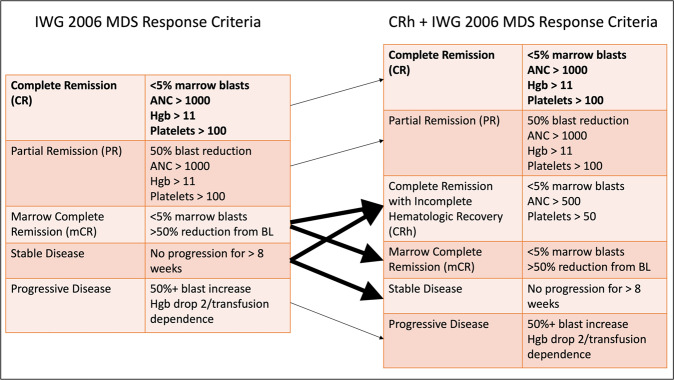
General classification of responses according to IWG 2006 response criteria (left) and when adding CRh as a response (right). Patients with marrow complete remission or stable disease were reclassified as CRh if, during therapy, they met the criteria for this metric.

We assessed initial responses to DNMTI therapy first according to IWG 2006 criteria. Because CRh is a combined marrow response (which incorporates both marrow responses as well as hematologic parameters), for this comparison we focused on the IWG 2006 marrow responses separate from hematologic improvement, understanding the latter is already associated with improved patient outcomes [17]. Among the 331 patients treated with azacitidine or decitabine, bone marrow responses included CR (*n* = 43, 14%), PR (*n* = 2, 1%), mCR (*n* = 57, 18%), SD (*n* = 149, 48%) and PD (*n* = 60, 19%) (Table 2). In addition to these responses, a total of 79 patients (25% of total) achieved some form of HI. We then reclassified patients who had mCR or SD as their best overall response to DNMTI therapy according to whether this instead met CRh criteria (Fig. 1). We identified a total 62 patients (20%) who met CRh criteria as a best overall response during DNMTI therapy. This resulted in concordant decreases in the number classified as mCR (*n* = 26, 8%), and SD (*n* = 118, 38%).

**Table 2:** Responses to azacitidine or decitabine according to IWG 2006 response criteria or modified criteria to include CRh.

IWG response	
CR	43 (14%)
PR	2 (1%)
mCR	57 (18%)
SD	149 (48%)
PD	60 (19%)
CRh response included	
CR	43 (14%)
PR	2 (1%)
CRh	62 (20%)
mCR	26 (8%)
SD	118 (38%)
PD	60 (19%)

We compared the characteristics of patients now classified as CRh responders to those who remained SD or mCR. The median age for patients with CRh was 69 years (range 45–86) compared to 68.5 for mCR/SD (range 23–91) (*p* = 0.58). There was no difference in baseline hemoglobin with a median of 9.6 g/dL in CRh compared to 9.2 g/dL for mCR/SD (*p* = 0.75), although median platelet count at baseline was higher at 104k in CRh compared to 71k in mCR/SD (*p* = 0.01) and the median baseline ANC of 1790/μL was CRh compared to 1240/uL for mCR/SD (*p* = 0.05). BM blasts were median 3.5% in CRh compared to 5.5% in mCR/SD (*p* = 0.03). Although there were some differences in baseline features as noted, the distribution in IPSS-R scores between CRh and mCR/SD groups was not significantly different (*p* = 0.07). Univariate comparisons between baseline the baseline characteristics of the patients who eventually achieved a response—CR, CRh, or either mCR or SD—are also shown in Table 1 and incorporated into the multivariate comparison.

Patients who have clinical benefit during DNMTI therapy may be expected to be more likely to continue with this treatment; we explored whether CRh may be a surrogate for “clinical benefit” by assessing the duration of time on HMA therapy according to modified response criteria. Patients achieving CR had the longest duration of time on HMA therapy (median 8.1mo) although this was not significantly longer than patients who achieved CRh (median 6mo, HR for discontinuing therapy 1.4, 95% CI 0.9–2.0). Patients in CR did however have a longer duration of therapy compared to other responses: mCR (median duration of therapy 4.7mo, HR for discontinuation 1.9 [1.2–3.1]), SD (median 4mo, HR 2.0 [1.4–2.9]), PR (median 4.1mo, HR 3.3 [0.8–13.8]) and PD (median 2.9mo, HR 5.0 [3.3–7.7]) (*p* < 0.001).

**Fig. 2:**
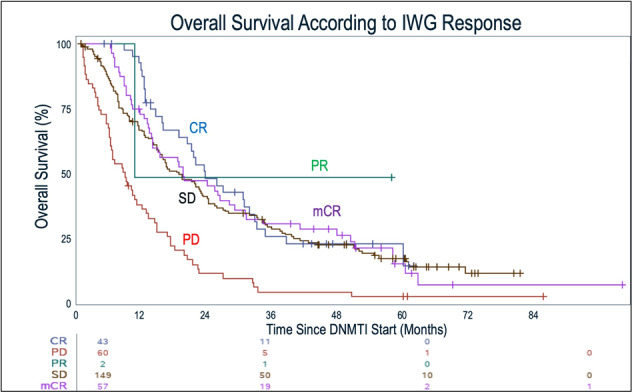
Overall survival from the time of therapy initial until death or last known alive, among patients treated with DNMTI therapy. Survival is shown according to the best overall response on DNMTI therapy (IWG 2006 criteria). Patients with CR as the BOR had prolonged survival compared to mCR, SD, and PD (*p* < 0.001).

**Fig. 3:**
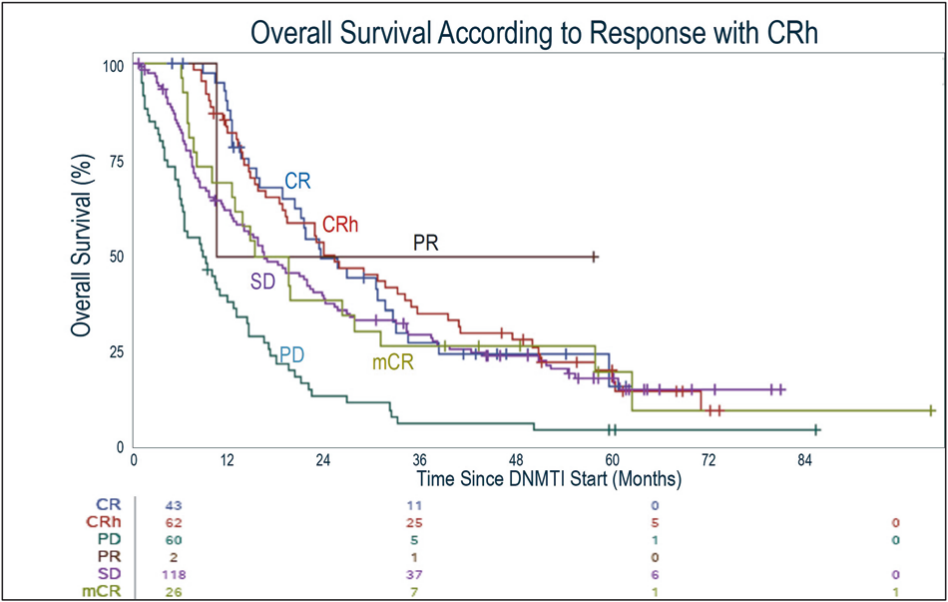
Overall survival from the time of therapy initial until death or last known alive, among patients treated with DNMTI therapy. Survival is shown according to the best overall response on DNMTI therapy (IWG 2006 criteria) but including patients who met CRh and reclassifying them from patients who met mCR or SD. Median OS was similar between CR (23.3mo) and CRh (25mo). This was longer than patients whose best response remained classified as mCR (17.2mo), SD (16.3mo), and PD (8.7mo) (*p* < 0.001).

We then assessed overall survival (OS) according to response on DNMTI therapy. First, we evaluated standard IWG 2006 responses, and confirmed prior studies [15–17] which showed that patients in this cohort whose best overall response was CR had improved OS compared to other IWG 2006 response outcomes (Fig. 2, median 23.3mo, *p* < 0.001). We subsequently evaluated survival according to responses and including CRh as an outcome, with receipt of allogeneic transplant as a time-varying covariate. Again, OS differed according to treatment response; the median OS was similar between those patients who achieved CR (23.3mo) and those patients who now had a response classified as CRh (25mo, HR 1.28 [0.79–2.08]) (Fig. 3). Patients who achieved CR had a longer survival compared to those whose best overall responses remained mCR (17.2mo, HR 1.71 [0.96–3.05]) and SD (16.3mo, HR 1.61 [1.04–2.48]) (excluding the patients who now met CRh), and were also improved compared to the OS of patients with disease progression on DNMTI therapy (median OS 8.7mo, HR 3.04 [1.91–4.83]) (*p* < 0.001). Other variables associated with OS on univariate analysis included IPSS-R risk score (*p* < 0.001), while WHO subgroup (*p* = 0.12) and DNMTI choice (*p* = 0.65) were not associated with survival. Given some differences in baseline characteristics, we performed a multivariate analysis including age, WHO classification, IPSS-R risk group, treatment with azacitidine or decitabine, allogeneic transplant as a time-varying covariate, and best overall response including CRh. Overall survival was associated with lower age (*p* = 0.024) and IPSS-R group (*p* < 0.0001) (Table 3). As expected, allogeneic transplant was also associated with significantly improved OS (*p* < 0.0001, HR 0.44, 95% CI 0.30–0.64). Accounting for these variables, we also found that best overall response was associated with improvement in survival as well (*p* < 0.0001). In this model, with CR as the reference, there was no significant difference in survival comparing CRh to CR (HR for mortality 1.28, 95% CI 0.79–2.08, *p* = 0.32). This finding was similar when we restricted the multivariate analysis to only patients with intermediate, high, and very-high IPSS-R risk disease (HR 1.29, 95% CI 0.72–2.33, *p* = 0.39). Similarly, baseline ANC, hemoglobin, platelet count, and bone marrow blast count did not impact our findings when accounting for the IPSS-R score.

**Table 3:** Multivariate analysis for overall survival.

Variable		*p* value	Hazard ratio	95% Hazard ratio confidence limits	
Allogeneic transplant^a^		<0.0001	0.44	0.304	0.636
Age		0.0236	1.018	1.002	1.034
WHO subgroup	MDS with Del5q	1.0			
	MDS NOS	0.3825	1.999	0.422	9.468
	MDS-EB1	0.3056	2.12	0.504	8.921
	MDS-EB2	0.3455	2.001	0.474	8.457
	MDS-MLD	0.4139	1.819	0.433	7.637
	MDS-SLD	0.3733	2.022	0.429	9.522
IPSS-R	Very low risk	1.0			
	Low risk	0.787	1.087	0.595	1.986
	Intermediate risk	0.9906	1.004	0.546	1.844
	High risk	0.1055	1.674	0.897	3.124
	Very high risk	<0.0001	3.611	1.971	6.613
Treatment	Azacitidine	0.1592	1.259	0.914	1.736
Best overall response	CR		1.0		
	CRh	0.3156	1.281	0.79	2.077
	SD	0.033	1.605	1.039	2.48
	mCR	0.0686	1.712	0.96	3.052
	PR	0.9494	1.067	0.142	8.016
	PD	<0.0001	3.038	1.91	4.831

^a^Allogeneic transplant status was considered as a time-varying covariate.

## Discussion

Uniform response criteria are important for patient clinical management, prognostication, and determination of active therapeutic agents for patients with myelodysplastic syndromes. The most commonly utilized response criteria in higher-risk MDS are the 2006 proposal by the international working group; [12–14] however, in practice, these criteria have a number of limitations, including arbitrary cutoffs between responses and vague definitions that limit reproducibility or comparisons between trials [11, 20]. Nonetheless, several studies have validated these response criteria as predicting overall survival [15, 16], particularly responses that are associated with hematologic benefit [17], making them important early surrogates for therapeutic activity. Notably, CR has been considered by the FDA as an potential approval endpoint in patients with MDS, including in ongoing pivotal phase 3 studies, although to date CRh has not been incorporated as a response.

Practically, the application of 2006 IWG response criteria creates a number of challenges both in the assessment of clinical trials as well as in the direct care of patients with MDS [21, 22]. Only a minority of patients will actually achieve a CR, while the majority of patients will have markedly heterogeneous responses to therapy captured as mCR or SD and with or without varying degrees of hematologic improvement. Even hematologic improvement can be challenging regarding its clinical impact; a patient may experience isolated neutrophil improvement, for instance, yet remain heavily transfusion dependent, yet be considered to have the same response as a patient without transfusion needs on therapy. Moreover, the need to assess marrow responses and hematologic responses at times in concert (CR, PR) and at times separately (mCR, SD, with or without HI) lead to duplication of patients when reporting clinical trials and are unnecessarily cumbersome in practice [11].

CRh was identified as a response metric first in acute lymphoblastic lymphoma with a study evaluating the activity of blinatumomab [23], and subsequently has been used to report activity in other acute leukemias [18, 24, 25]. The rationale for this metric stems in part from the ability to assess measurable residual disease in acute leukemias by flow cytometric or molecular methods; [26, 27] patients who are without detectable disease but nonetheless have reasonable count improvement—an ANC greater than 500/μL and platelet count greater than 50 k/μL, levels at which infectious and bleeding complications decline—may represent a clinically meaningful group who can proceed to further therapies such as further consolidation cycles or transplant. Although MRD assessment in MDS is complex and as of yet investigational, CRh does encompass several clinically meaningful “goals” in MDS therapy. Of note, CRh notably does not address RBC transfusion needs and anemia, although such is perhaps most relevant during lower risk MDS management.

The use of CRh in MDS, in this sense, serves as an example to challenge whether there may be alternative, meaningful responses to evaluate when treating patients with MDS. The criteria for CRh have reasonable clinical relevance in that they represent common metrics—an ANC above 500 and a platelet count above 50k both are associated with lower patient-specific risks—and also incorporate a reduction in blast count. Of course, these cut-offs are also arbitrary, as it is not clear that a platelet count of 40k or ANC of 400 would be any worse for the patient. In addition, red cell transfusion needs or hemoglobin levels are meaningful in MDS; ideally, larger studies could assess response cut-offs and then prospectively validate them. Nonetheless, we felt that CRh represents an example of a combined marrow and blood count metric from which further assessment could begin.

Indeed, we confirmed that CR remained a robust clinical response metric for patients with MDS receiving DNMTI therapy, and remains a valuable predictor of OS. At the same time, we found that patients with MDS who achieve a CRh equivalent response had a similar duration on therapy as well as overall survival to those who achieved the stricter metric of CR. These patients also had improved outcomes compared to those with other marrow responses. Indeed, this identified a subgroup representing 20% of all patients with overlapping survival benefit, and CR + CRh together encompassed 34% of the entire cohort. It is important to note that there were some characteristics that were potentially more favorable among the patients who eventually achieved CRh compared to those that remained classified as mCR or SD, including baseline blood counts and blasts, and larger cohorts are needed to evaluate this and other novel response criteria. Multivariate analysis suggested that CRh responses were similar to CR responses, even when accounting for some of these baseline variables, but may be underpowered to detect small differences in this cohort. Indeed, when we divided patients by IPSS-R risk into lower (very low and low) and higher (intermediate, high, and very high) risk cohorts, most of the survival difference seemed to be restricted to the higher risk group (Supplementary Fig. S1). Nonetheless, for clinical care and for trial interpretation, we feel that this analysis illustrates the role that integrated response criteria may have to distinguish best responders on a novel therapy or combination.

In conclusion, these data support the further evaluation of CRh or other combined marrow/blood count response endpoints into future iterations of response criteria in MDS. These data also suggest new endpoints which could be evaluated in ongoing prospective studies, such as those evaluating novel DNMTI doublets. While some IWG response metrics continue to demonstrate clear clinical and research value, we feel that this data suggests ways in which we could meaningfully refine responses to optimize patient outcomes in this malignancy.

## Supplementary information

Supplemental Figure S1

## Data Availability

Please contact the corresponding author for data requests.
