# Supplementary material for: Evaluating complete remission with partial hematologic recovery (CRh) as a response criterion in myelodysplastic syndromes (MDS)
Source: Blood Cancer J. 2022 Nov 15;12(11):153. doi: 10.1038/s41408-022-00748-9 (PMC9666661; doi:10.1038/s41408-022-00748-9)

Supplementary Data

Figure S1: survival according to IWG 2006 and IWG 2006+CRh in patients with IPSSR INT, HIGH, and VERY HIGH risk MDS

Figure S1A. Survival of patients with intermediate, high, and very high IPSS-R risk MDS according to response. Patients with CRh had similar survival to CR and improved compared to other groups. P<0.01.


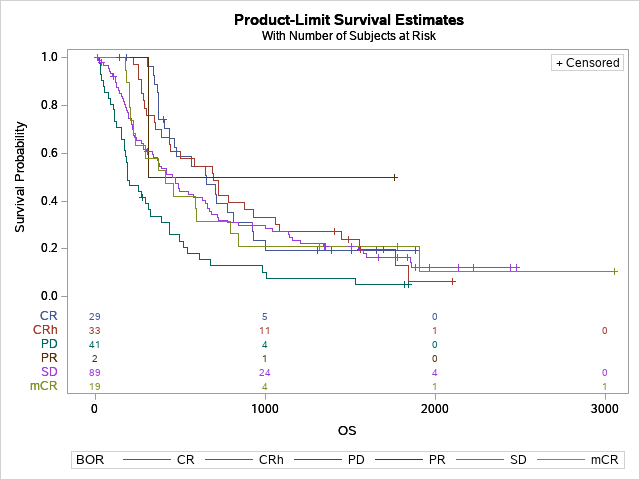


Figure S1B. Survival of patients with low and very low IPSS-r risk MDS according to response. There was still a difference in survival according to response, but related to patients with progressive disease (P=0.03).


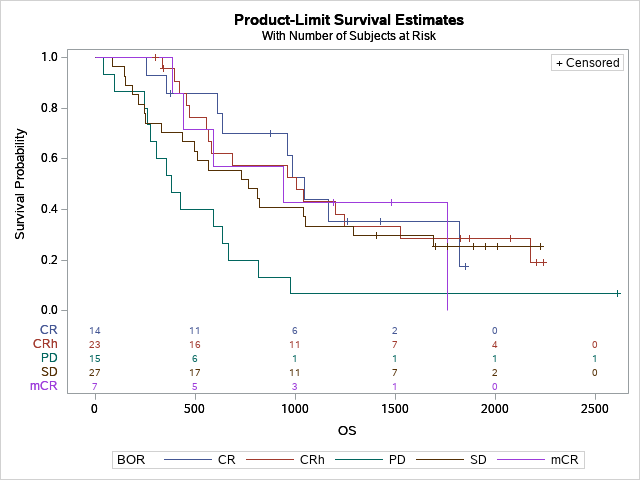

Supplement: Supplementary file 1 — Supplemental Figure S1 [file 41408_2022_748_MOESM1_ESM.docx]
